# Decreased expression of microRNA-17 and microRNA-20b promotes breast cancer resistance to taxol therapy by upregulation of NCOA3

**DOI:** 10.1038/cddis.2016.367

**Published:** 2016-11-10

**Authors:** Xiang Ao, Peipei Nie, Baoyan Wu, Wei Xu, Tao Zhang, Songmao Wang, Haocai Chang, Zhengzhi Zou

**Affiliations:** 1 Breast Oncology Department, Cancer Center of Guangzhou Medical University, Guangzhou, Guangdong, China; 2 KingMed Diagnostics and KingMed School of Laboratory Medicine, Guangzhou Medical University, Guangzhou, Guangdong, China; 3 MOE Key Laboratory of Laser Life Science and Institute of Laser Life Science, College of Biophotonics, South China Normal University, Guangzhou, Guangdong, China

## Abstract

Chemoresistance is a major obstacle to effective breast cancer chemotherapy. However, the underlying molecular mechanisms remain unclear. In this study, nuclear receptor coactivator 3 (NCOA3) was found to be significantly increased in taxol-resistant breast cancer tissues and cells. Moreover, overexpression of NCOA3 enhanced breast cancer cell resistance to taxol, whereas depletion of NCOA3 decreased taxol resistance. Subsequently, we investigated whether NCOA3 expression was regulated by miRNAs in breast cancer. By bioinformatics prediction in combination with the data of previous report, miR-17 and miR-20b were selected as the potential miRNAs targeting NCOA3. By real-time PCR analysis, we found that miR-17 and miR-20b were significantly reduced in taxol-resistant breast cancer tissues and cells. In addition, we provided some experimental evidences that miR-17 and miR-20b attenuated breast cancer resistance to taxol *in vitro* and *in vivo* models. Furthermore, by luciferase reporter assays, we further validated that both miR-17 and miR-20b directly binded the 3′-untranslated region of *NCOA3* mRNA and inhibited its expression in breast cancer cells. Finally, both miR-17 and miR-20b levels were found to be significantly negatively correlated with *NCOA3* mRNA levels in breast cancer tissues. Together, our results indicated that loss of miR-17 and miR-20b enhanced breast cancer resistance to taxol by upregulating NCOA3 levels. Our study suggested miR-17, miR-20b and NCOA3 may serve as some predictive biomarkers and potential therapeutic targets in taxol-resistant breast cancer treatment.

Breast cancer is a very aggressive tumor with poor prognosis. Although taxol (paclitaxel) as an important chemotherapeutic agent in the treatment of breast cancer have shown promise, cancer cells resistance to taxol frequently results in the subsequent recurrence of cancer.^1^ The detailed molecular mechanisms involved with cancer cell resistance to taxol are still unknown. The first known mechanism is overexpression of the MDR-1 gene encoding P-glycoprotein in taxol-resistant cancer cells.^2^ P-glycoprotein functions as a drug-efflux pump on the surface of cancer cell to efflux taxol.^3^ Other mechanisms responsible for taxol resistance include the mutations of taxol-binding protein tubulin, deregulation of cell cycle and high expression of anti-apoptotic protein.^4^

Nuclear receptor coactivator 3 (NCOA3), also known as amplified in breast cancer 1 (AIB1) is a member of the p160/steroid receptor coactivator (SRC) family.^5^ NCOA3 as a nuclear receptor coactivator promotes breast cancer tumorigenesis and malignant progression by enhancing the transcriptional activity of estrogen receptor (ER) and progesterone receptor (PR).^6^ Multiple studies have also shown that NCOA3 enhances the activity of a number of other transcription factors, such as E2F-1, AP-1, NF-*κ*B and STAT6.^7^ NCOA3 has been found to have important role in a number of biological processes, such as cell proliferation, apoptosis and migration.^8, 9^ *NCOA3* gene amplification has been found in 2–10% of human breast cancer.^10^ Moreover, NCOA3 is overexpressed in 30–60% breast cancer and associated with high histological grade, advanced stage and poor prognosis.^10^ An oncogenic role of NCOA3 has also been found in other cancer types, such as prostate, ovarian, esophageal, gastric, colon and liver cancers.^9, 11^ Previous study show high expression of NCOA3 promote tamoxifen resistance by increasing ER activity in breast cancer.^12^ Whereas another study indicates that patients with high nuclear expression of NCOA3 tend to respond well to tamoxifen therapy.^13^ Moreover, overexpression of NCOA3 predicts resistance to chemoradiotherapy in esophageal squamous cell carcinoma.^14^ Our previous study suggests that overexpression of NCOA3 can suppress breast cancer cells apoptosis induced by histone deacetylase inhibitors.^8^

MicroRNAs (miRNAs), a class of 16- to 29-nucleotide-long single-stranded non-protein-coding RNAs that negatively regulate gene expression, have been shown to control several important biological processes, including cell proliferation, apoptosis, differentiation, invasion and migration.^15, 16, 17^ Numerous miRNAs are highly expressed or low expressed in tumor, contributing to the initiation and drugs resistance of the cancer. For instance, miRNA-451 high expression sensitizes breast cancer cells to adriamycin,^18^ whereas upregulation of miRNA-21 promote cancer cell resistance to trastuzumab.^19^ In addition, high-expressed miR-125b confers the resistance of breast cancer cells to taxol through inhibition of Bcl-2 and Bak1 expression.^20^ Moreover, recent study reports that miRNA-205 enhances breast cancer cell resistance to doxorubicin and taxol by targeting VEGFA and FGF2.^21^

MicroRNA-17 (miR-17) and microRNA-20b (miR-20b) belong to the miR-17-92 and miR-106a-363 clusters, respectively. The two clusters along with miR-106b-25 cluster form the miR-17 family. The expression of mature miR-17-92 cluster members is often substantially increased in human B-cell lymphomas and modulates tumor formation.^22^ MiR-17 has been found to be highly expressed in embryonic cells and has a vital role in embryonic development.^23^ Several studies have reported that miR-17 is significantly upregulated in gastric cancer, and promotes cells growth and migration.^24^ In contrast, another study finds miR-17 inhibits colorectal cancer progression by suppressing tumor angiogenesis.^25^ In addition, miR-20b as a potential oncogene has been reported to favor the survival of breast cancer cells upon the oxygen supply.^26^ Recent study found that high expression of miR-20b promotes breast cancer brain metastasis.^27^

In this study, *NCOA3* mRNA levels were found to be significantly increased in taxol-resistant breast cancer. Therefore, we investigated the relationship between NCOA3 with taxol resistance of breast cancer. We showed NCOA3 decreased breast cancer cell apoptosis induced by taxol. Subsequently, we investigated whether NCOA3 expression was regulated by miRNAs in breast cancer. By bioinformatics prediction in combination with the data of previous report, miR-17 and miR-20b were selected as two potential miRNAs targeting NCOA3. Next, we provided some experimental evidences that miR-17 and miR-20b attenuated breast cancer resistance to taxol *in vitro* and *in vivo* models. Furthermore, we validated that both miR-17 and miR-20b directly targeted and inhibited the expression of NCOA3 and therefore reduced taxol-induced cytotoxicity in breast cancer cells. Finally, both miR-17 and miR-20b levels were found to be significantly negatively correlated with *NCOA3* mRNA levels in breast cancer tissues.

## Results

### Overexpression of NCOA3 enhances breast cancer resistance to taxol

**Figure 1:**
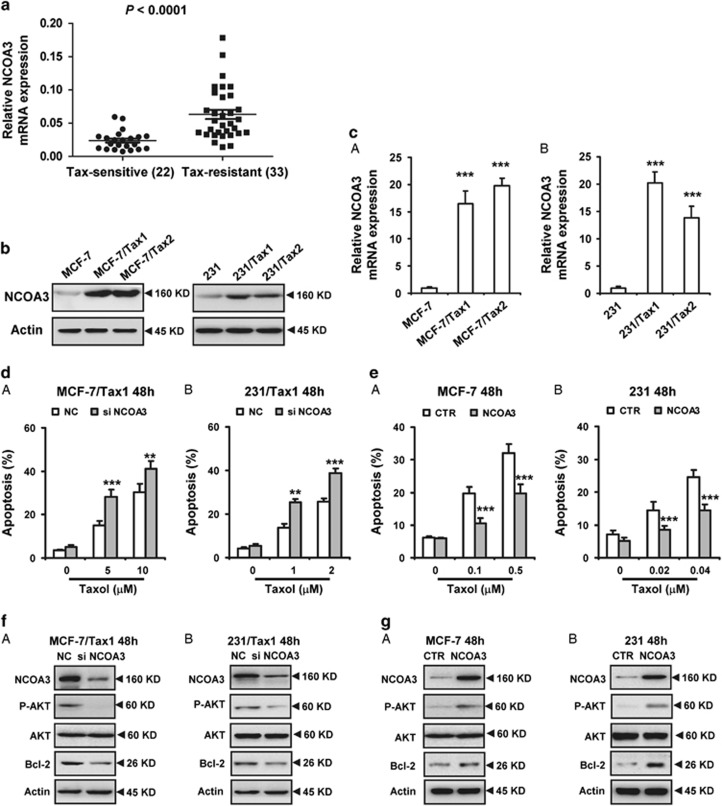
Overexpression of NCOA3 enhances breast cancer resistance to taxol. (**a**) RT-PCR analysis of relative NCOA3 expression in 22 breast cancer tissues from taxol-sensitive patients and 33 breast cancer tissues from taxol-resistant patients. (**b** and **c**) Western blot and RT-PCR were performed to detect the protein and mRNA expression of NCOA3 in taxol-resistant MCF-7 cells (MCF-7/Tax1 and MCF-7/Tax2) and taxol-resistant 231 cells (231/Tax1 and 231/Tax2). Data were from three independent experiments. Actin was used as a loading control. Data represent mean±S.D. ***P<0.001. (**d** and **e**) (**d**) MCF-7/Tax1 (A) and 231/Tax1 (B) cells were transfected with NC and NCOA3 siRNA. (**e**) MCF-7 (A) and 231 (B) cells were transfected with control (CTR) and NCOA3 vectors. After 8 h, cells were treated with indicated dose of Taxol (Tax) for additional 48 h. Cell apoptosis was assessed by Annexin-V-FITC/PI staining assay by flow cytometry. Columns, means of three determinations; bars, S.D.; **P<0.01; ***P<0.001, compared with control treated cells. (**f**) MCF-7/Tax1 (A) and 231/Tax1 (B) cells were transfected with NC and NCOA3 siRNA for 48 h. (**g**) MCF-7 (A) and 231 (B) cells were transfected with CTR and NCOA3 vectors for 48 h. Western blot was performed to detect the indicated protein expression. Data were from three independent experiments

To assess whether NCOA3 has a role in breast cancer resistance to taxol, real-time PCR (RT-PCR) was used to analyze NCOA3 expression in breast carcinoma specimens. A total of 22 specimens from patients with taxol sensitivity and 33 specimens from patients with taxol resistance were included in this study (Supplementary Table S1). Our results showed taxol-resistant breast cancer tissue specimens exhibited generally higher *NCOA3* mRNA levels compared with taxol-sensitive tissues (Figure 1a). In addition, we also detected the mRNA levels of NCOA3 in the adjacent normal tissues of above breast cancer tissues. As shown in Supplementary Figures S1A and B, *NCOA3* mRNA levels were significantly upregulated in both taxol-sensitive and taxol-resistant cancer tissues relative to their adjacent normal tissues (Supplementary Figures S1A and B).

In addition, we detected the expression of NCOA3 in several breast cancer cells lines. We found that there were relative low expression of NCOA3 in non-triple-negative breast cancer (non-TNBC) cell line MCF-7 and TNBC cell line MDA-MB-231 (231) (Supplementary Figure S2). Thus, these two cell lines with relative low NCOA3 levels were selected to model the development of acquired taxol resistance in patients, and evaluate whether NCOA3 was upregulated by taxol. MCF-7 and 231 cells were treated with low dose of taxol for 12 months to select the taxol-resistant cells (named MCF-7/Tax1, MCF-7/Tax2, 231/Tax1 and 231/Tax2). Consistent with the results from breast cancer tissues, higher protein and mRNA levels of *NCOA3* were found in taxol-resistant breast cancer cells compared with the taxol-sensitive parental cells (Figures 1b and c).

To investigate the role of NCOA3 in the sensitivity of breast cancer cells to taxol, MCF-7/Tax1 and 231/Tax1 cells were treated with NCOA3 small interfering RNA (siRNA), and then cell apoptosis induced by taxol was measured. As shown in Figure 1d, cell apoptosis induced by taxol was significantly increased by NCOA3 siRNAs. Meanwhile, downregulation of NCOA3 significantly decreased the levels of anti-apoptotic protein Bcl-2 and phosphated AKT in both MCF-7/Tax1 and 231/Tax1 cells (Figure 1f). By contrast, overexpression of NCOA3 attenuated taxol-induced cell apoptosis in MCF-7 and 231 cells (Figure 1e). In addition, forced expression of NCOA3 observably enhanced Bcl-2 and phosphated AKT expression in the two breast cancer cells (Figure 1g). In addition, we found depletion of NCOA3 decreased MCL-1 expression and induced BAX expression in MCF-7/Tax1 cells (Supplementary Figure S3). No significant change in the BCL-XL level was found in MCF-7/Tax1 cells treated with NCOA3 siRNA (Supplementary Figure S3). These results suggested NCOA3 enhanced breast cancer cells resistance to taxol involved in downregulation of BAX and upregulation of Bcl-2, MCL-1 and AKT1 activation.

### MiR-17 and miR-20b are predicted to target NCOA3 and decreased in taxol-resistant breast cancer

**Figure 2:**
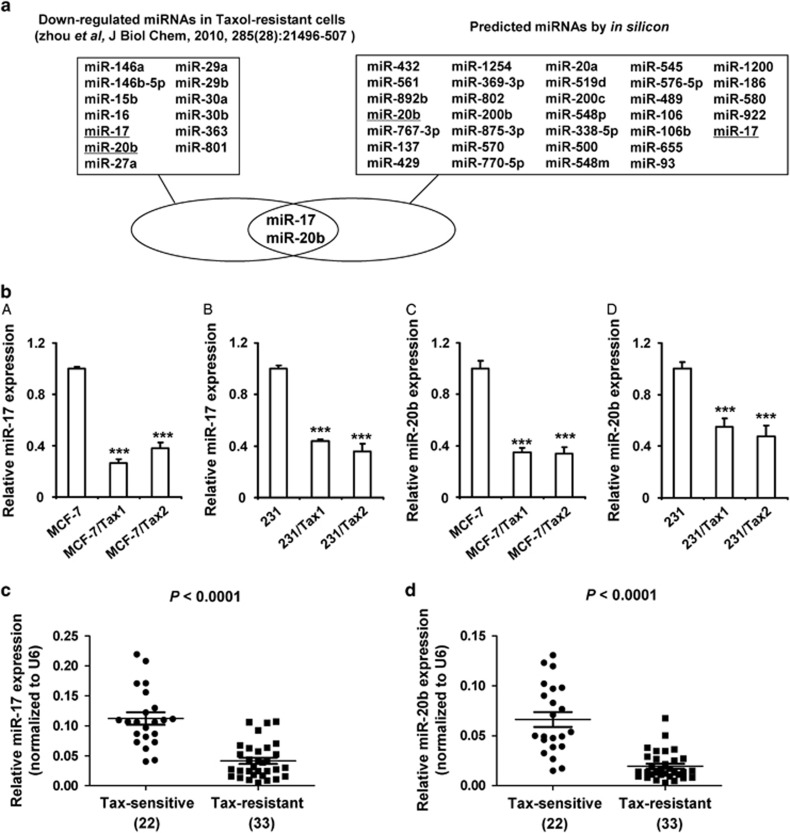
MiR-17 and miR-20b are predicted to target NCOA3 and decreased in taxol-resistant breast cancer. (**a**) Flowchart for the selection of the miR-17 and miR-20b. (**b**) RT-PCR was performed to detect the expression of miR-17 and miR-20b in MCF-7, 231, MCF-7/Tax1, MCF-7/Tax2, 231/Tax1 and 231/Tax2 cells. U6 was used as an internal control. Data represent mean±S.D. ***P<0.001. (**c** and **d**) RT-PCR were performed to detect relative expression of miR-17 and miR-20b in 22 breast cancer tissues from taxol-sensitive patients and 33 breast cancer tissues from taxol-resistant patients. U6 was used as an internal control. Data represent mean±S.D. *P*<0.0001

To investigate whether downregulation of miRNAs increased NCOA3 expression in taxol-resistant breast cancer, we carried out a bioinformatic prediction to search miRNAs targeting the 3′-untranslated region (3′-UTR) of *NCOA3*. We initially predicted 33 shared target miRNAs of NCOA3 in four online databases (Figure 2a). To narrow this list of potential miRNA regulators of NCOA3 in taxol-resistant breast cancer, we analyzed the only data set of publically available miRNA array data from taxol-resistant breast cancer.^20^ Only 13 miRNAs are reported be significantly reduced in taxol-resistant breast cancer cells (Figure 2a). Based on the 33 predicted target miRNAs and 13 reported miRNAs, miR-17 and miR-20b were selected for further analysis. The expression levels of miR-17 and miR-20b were evaluated by RT-PCR in taxol-resistant breast cancer cells. As shown in Figure 2b, the levels of both miRNAs were significantly decreased in taxol-resistant breast cancer cell lines (MCF-7/Tax1, MCF-7/Tax2, 231/Tax1 and 231/Tax2) compared with their parent cell lines (MCF-7 and 231).

To further examine whether miR-17 and miR-20b levels were decreased in taxol-resistant breast cancer, we analyzed miR-17 and miR-20b levels in 22 breast cancer tissues from taxol-sensitive patients and 33 breast cancer tissues from patients with taxol resistance. As shown in Figure 2c, the levels of both miRNAs were observably decreased in taxol-resistant breast cancer specimens. Our results were consistent with the results reported in previous study.^20^ In addition, we also detected the expression of miR-17 and miR-20b in the adjacent normal tissues of above breast cancer tissues. In taxol-sensitive patients, both miRNA levels were shown no significant difference in breast cancer tissues and their adjacent normal tissues (Supplementary Figures S4A and B). Notably, in taxol-resistant patients, tumor tissues exhibited generally lower miR-17 and miR-20b levels compared with their adjacent normal tissues (Supplementary Figures S4C and D).

### Both miR-17 and miR-20b enhance taxol-induced apoptosis in breast cancer cells

**Figure 3:**
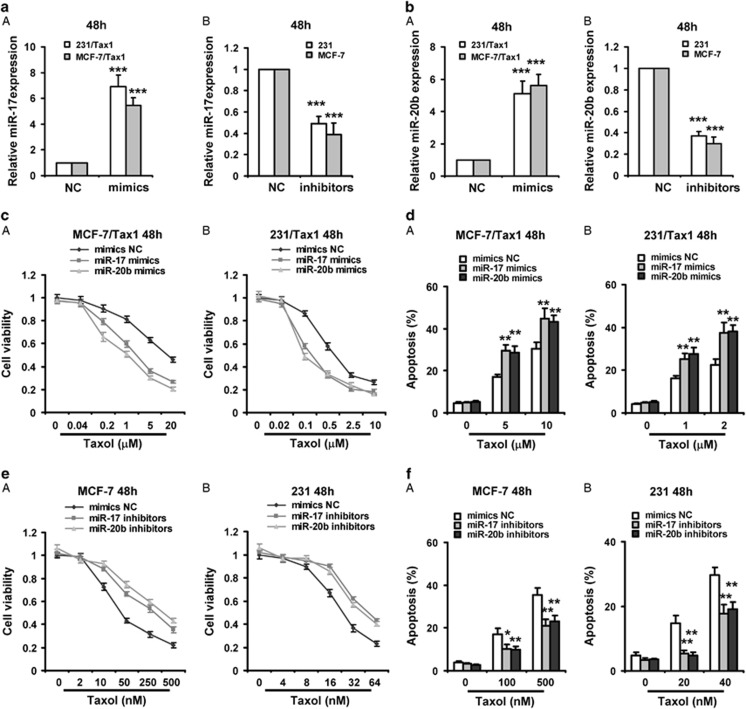
Both miR-17 and miR-20b enhances taxol-induced apoptosis in breast cancer cells. (**a** and **b**) (a) 231/Tax1 and MCF-7/Tax1 were transfected with miR-17 mimics (**a**) or miR-20b mimics (**b**) and NC for 48 h, respectively. (B) MCF-7 and 231 cells were transfected with miR-17 inhibitors (**a**) or miR-20b inhibitors (**b**) and NC for 48 h, respectively. RT-PCR was performed to detect the expression of miR-17 or miR-20b. (**c** and **d**) MCF-7/Tax1 (A) and 231/Tax1 (B) were transfected with miR-17 or miR-20b mimics. (**e** and **f**) MCF-7 (A) and 231 (B) were transfected with miR-17 or miR-20b inhibitors. After 8 h, cells were treated with indicated dose of taxol (Tax) for additional 48 h. MTT assay was performed to examine cell viability (**c** and **e**). Cell apoptosis was assessed by Annexin-V-FITC/PI staining assay by flow cytometry (**d** and **f**). Columns, means of three determinations; bars, S.D. *P<0.05; **P<0.01; ***P<0.001, compared with NC-treated cells

To investigate the roles of miR-17 and miR-20b in the sensitivity of breast cancer cells to taxol, MCF-7/Tax1 and 231/Tax1 were treated with miR-17 or miR-20b mimics, and the cytotoxicity induced by taxol was examined. As shown in Figures 3aA and Ba, forced expression of miR-17 and miR-20b mimics in both taxol-resistant cancer cells significantly increased miR-17 and miR-20b levels, respectively. Moreover, both miRNA mimics significantly increased the cytotoxicity of taxol and taxol-induced cell apoptosis in both taxol-resistant breast cancer cells (Figures 3c and d). On the contrary, miR-17 and miR-20b inhibitors apparently decreased miR-17 and miR-20b levels in MCF-7 and 231 cells (Figures 3aB and bB). Moreover, downregulation of miR-17 and miR-20b by miRNA inhibitors observably reduced cell toxicity of taxol and taxol-induced cell apoptosis in both MCF-7 and 231 cells (Figures 3e and f). In addition, by sub-G1 assays, we also showed both miR-17 and miR-20b mimics significantly increased taxol-induced cell apoptosis in both taxol-resistant breast cancer cells (Supplementary Figures S5A, B and C), whereas both miRNAs inhibitors observably reduced taxol-induced cell apoptosis in both MCF-7 and 231 cells (Supplementary Figures S5D and E).

The caspase 3 has been implicated as a key mediator of apoptosis in mammalian cells. To test whether miR-17 and miR-20b increased taxol-induced caspase 3 activities, we detected caspase 3 activities by fluorogenic substrate cleavage assays in breast cancer cells after treatment with miRNA mimics or inhibitors plus taxol. Our results indicated that miR-17 and miR-20b mimics observably increased caspase 3 activities in MCF-7/Tax1 and 231/Tax1 cells (Supplementary Figures S6A and B), whereas both miRNAs inhibitors significantly decreased caspase 3 activities in MCF-7 and 231 cells (Supplementary Figures S6C and D).

### Identification of NCOA3 as a direct target of miR-17 and miR-20b

**Figure 4:**
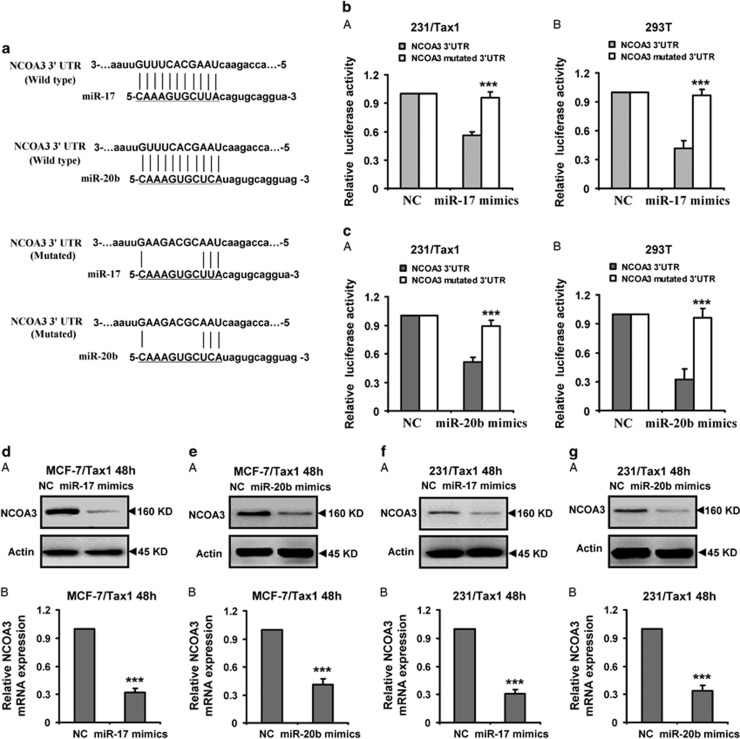
Identification of NCOA3 as a direct target of miR-17 and miR-20b. (**a**) The predicted miR-17 and miR-20b target sites in the 3′-UTR of *NCOA3* mRNA and their mutated version. (**b** and **c**) Luciferase activity assays in 231/Tax1 and 293T cells showed that miR-17 and miR-20b inhibited the expression of *NCOA3*. 231/Tax1 and 293T cells were co-transfected with pGL3 vector containing the wild-type or mutated 3′-UTR of *NCOA3*, or pGL3-control vector, along with miR-17 or miR-20b mimics and NC. After 48 h, luciferase activity was detected. Data were normalized to luciferase activity in the corresponding cells transfected with NC and are represented as the mean±S.D. of three replicates. (**d**-**g**) Protein and mRNA levels of NCOA3 were downregulated by miR-17 or miR-20b mimics in breast cancer cells. MCF-7/Tax1 (**d** and **e**) and 231/Tax1 (**f** and **g**) cells were transfected with miR-17 or miR-20b mimics and NC, respectively. (A) Western blot was performed to detect the protein expression of NCOA3. Actin was used as a loading control. Data were from three independent experiments. (B) RT-PCR was performed to detect the mRNA expression of NCOA3. Actin was used as control. Data are mean±S.D. from three independent experiments. ***P<0.001, compared with the control group

To further validate the targeting of NCOA3 by miR-17 and miR-20b, luciferase activity assay was performed. The potential binding sites of miR-17 and miR-20b were shown in Figure 4a. The wild-type and mutated 3′-UTRs of *NCOA3* were cloned into the downstream of firefly luciferase coding region in pGL3 luciferase reporter vector respectively (Figure 4a). The vectors were co-transfected with miR-17 or miR-20b mimics into 231/Tax1 or 293T cells, respectively. As expected, the two miRNA mimics significantly decreased luciferase activity in 231/Tax1 or 293T cells transfected wild-type reporter vectors (Figures 4b and c). However, no obvious reduction of luciferase activities by both miR-17 and miR-20b mimics were observed in 231/Tax1 or 293T cells transfected mutated type reporter vectors (Figures 4b and c). Moreover, the protein and mRNA levels of *NCOA3* were detected in MCF-7/Tax1 and 231/Tax1 cells transfected miR-17 or miR-20b mimics. As shown in Figures 4d and g, both miR-17 and miR-20b mimics significantly decreased protein and mRNA expression of *NCOA3* in the two cells. Above results implied that miR-17 and miR-20b suppressed the NCOA3 expression by binding 3′-UTR of the gene mRNA.

### MiR-17 and miR-20b are negatively correlated with NCOA3 mRNA levels in breast cancer

**Figure 5:**
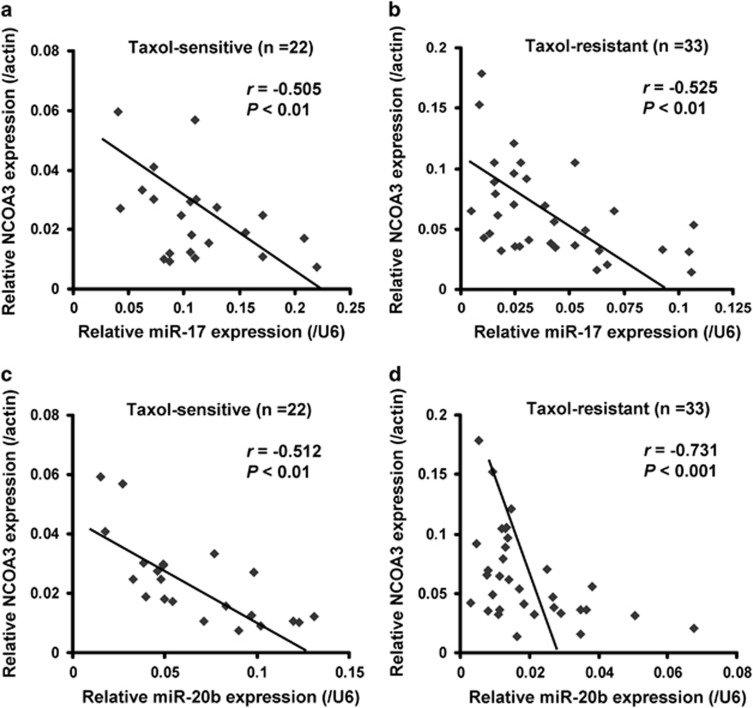
MiR-17 and miR-20b are negatively correlated with NCOA3 mRNA levels in breast cancer. Relative expression of NCOA3 along with miR-17 and miR-20b was determined by RT-PCR in 22 taxol-sensitive and 33 taxol-resistant breast cancer tissues from patients with taxol treatment. (**a**-**d**) Relative expression of NCOA3 along with miR-17 and miR-20b were determined by RT-PCR in 22 taxol-sensitive breast cancer tissues (**a** and **c**) and 33 taxol-resistant breast cancer tissues (**b** and **d**). For NCOA3, *β*-Actin was used as an internal control; For miR-17 and miR-20b, U6 was used as an internal control. Their expression correlations were analyzed by correlation coefficient and *t*-test

To further examine whether miR-17 and miR-20b levels were correlated with NCOA3, miR-17 and miR-20b along with *NCOA3* mRNA levels were analyzed in 22 breast cancer tissues from patients with taxol sensitivity and 33 breast cancer tissues from patients with taxol resistance. By correlation analysis between miR-17 and NCOA3, we found significantly negative correlations between miR-17 and *NCOA3* mRNA expression in both taxol-sensitive specimens (*r*=−0.505, *P*<0.01; Figure 5a) and taxol-resistant specimens (*r*=−0.525, *P*<0.01; Figure 5b). In addition, correlation analysis was also performed between miR-20b and NCOA3 in the two types of specimens. Similarly, significantly negative correlations between miR-20b and *NCOA3* mRNA expression levels were found in both taxol-sensitive specimens (*r*=−0.512, *P*<0.01; Figure 5c) and taxol-resistant specimens (*r*=−0.731, *P*<0.001; Figure 5d). These results raised a possibility that the expression of NCOA3 was suppressed by both miR-17 and miR-20b *in vivo.*

### Overexpression of NCOA3 reverses the attenuation of tamoxifen resistance by miR-17 and miR-20b in breast cancer cells

**Figure 6:**
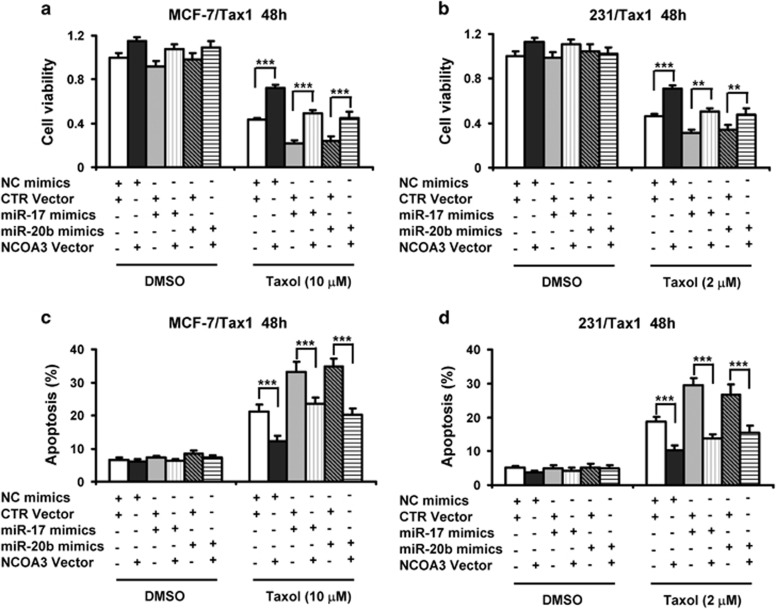
Overexpression of NCOA3 reverses reduction of cell viability and induction of apoptosis by miR-17 and miR-20b in taxol-treated breast cancer cells. (**a**-**d**) MCF-7/Tax1 (**a** and **c**) and 231/Tax1 (**b** and **d**) cells were co-transfected with NC and miR-17 or miR-20b mimics along with control (CTR) or NCOA3 vectors. After 8 h, cells were treated with indicated dose of taxol (Tax) for additional 48 h. (**a** and **b**) MTT assay was performed to examine cell viability. (**c** and **d**) Cell apoptosis was assessed by Annexin-V-FITC/PI staining assay by flow cytometry. Columns, means of three determinations; bars, S.D.; **P<0.01; ***P<0.001, compared with cells treated by NC plus CTR

To further determine that miR-17 and miR-20b attenuated breast cancer cell resistance to taxol was involved in repressing NCOA3, MCF-7/Tax1 and 231/Tax1 cells were co-transfected with miR-17 or miR-20b mimics along with NCOA3 overexpression constructs, subsequently cells were treated with taxol. Supplementary Figures S7A and B showed NCOA3 overexpression vectors distinctly increased the mRNA levels of *NCOA3* in MCF-7/Tax1 and 231/Tax1 cells. Moreover, the attenuation of *NCOA3* mRNA levels by miR-17 and miR-20b was remarkably increased by NCOA3 overexpression vectors (Supplementary Figures S7A and B). On the other hand, taxol-inhibited cell viability was significantly decreased by both miR-17 and miR-20b in MCF-7/Tax1 and 231/Tax1 cells, whereas NCOA3 overexpression evidently blocked taxol-induced cytotoxicity (Figures 6a and b). More importantly, the attenuation of breast cancer cell resistance to taxol by miR-17 and miR-20b were significantly reversed by NCOA3 overexpression (Figures 6a and b). In addition, we showed taxol-induced cell apoptosis was markedly increased by both miR-17 and miR-20b in MCF-7/Tax1 and 231/Tax1 cells, whereas NCOA3 overexpression significantly reduced taxol-induced cell apoptosis (Figures 6c and d). Moreover, the increase of taxol-induced cell apoptosis by miR-17 and miR-20b were significantly reversed by NCOA3 overexpression (Figures 6c and d).

### MiR-17 and miR-20b decrease taxol resistance in xenograft breast tumor models

**Figure 7:**
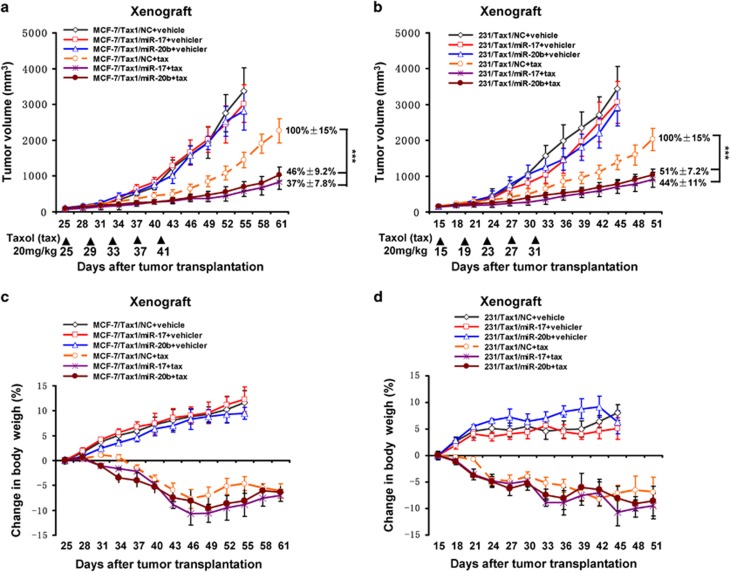
MiR-17 and miR-20b decrease resistance of breast tumor to taxol in xenograft tumor models. MCF-7/Tax1 and 231/Tax1 cells stably expressing miR-17 or miR-20b mimics or NC mimics were injected into nude mice. Nude mice were administered with taxol (20 mg/kg) as indicated at each time point. (**a** and **b**) Tumor volume was measured once per three days by using calipers (as indicated at each time point) for 30–36 days. (**c** and **d**) Average body weight changes were measured over the course of the study. Data are shown as mean±S.D. (*n*=6 per group). ***P<0.001

To investigate whether miR-17 and miR-20b enhanced sensitivity of breast tumor to taxol *in vivo*, MCF-7/Tax1 and 231/Tax1 cells stably expressing miR-17 and miR-20b were injected subcutaneously into female nude mice. Tumors were allowed to grow for 15 or 25 days. Subsequently, the nude mice were administered with taxol, and the volumes of tumors as well as body weight of mice were measured. As shown in Figures 7a and b, we showed that miR-17 and miR-20b had no effect on tumor growth in the mice without taxol treatment. After administration with taxol, the tumors originating from cells expressing miR-17 and miR-20b were almost twofold smaller in size than those tumors originating from control cells (Figures 7a and b). These results suggested that both miR-17 and miR-20b significantly decreased breast cancer cells resistance to taxol *in vivo*. Notably, after taxol treatment, no significant differences of body weight were found between mice bearing tumors originating from taxol-resistant breast cancer cells stably expressing miR-17 or miR-20b and mice bearing tumors originating from control cells (Figures 7c and d).

## Discussion

Here, we showed for the first time that NCOA3 is upregulated in breast cancer tissues derived from taxol-resistant patients, compared with the tumor samples from taxol-sensitive patients. In addition, we verified that NCOA3 was also upregulated in taxol-resistant MCF-7 and 231 breast cancer cells relative to their parental cells. Moreover, consistent with previous studies,^5^ the expression of NCOA3 was significantly increased in all primary tumors analyzed (i.e., taxol-resistant and taxol-sensitive tumor tissues) relative to their adjacent normal tissues.

NCOA3 as a transcriptional coregulator regulates cancer cell functions by directly interacting with nuclear receptors and other transcription factors, and enhancing their transcriptional activities.^7^ In this study, by ectopic expression and loss-of-function experiments of NCOA3, we indicated NCOA3 enhanced breast cancer cells resistance to taxol. Moreover, we found that NCOA3 led to marked upregulation of Bcl-2, which blocks apoptosis by suppressing mitochondrial permeability. Previous study shows that NCOA3 acts as a transcriptional coactivator of NF-*κ*B and upregulates NF-*κ*B signaling in cancer cells.^7^ Activated NF-*κ*B directly induces the transcription of Bcl-2 gene.^28^ Therefore, these results raised the possibility that NCOA3 enhanced the expression of Bcl-2 through NF-*κ*B/Bcl-2 pathway. In addition, our results showed that NCOA3 induced the activation of AKT. Several investigations indicated that NCOA3 increases insulin-like growth factor-I (IGF-I) and epidermal growth factor (EGF) signaling, known upstream regulation signaling of AKT.^29^ It has been shown that AKT confers drugs resistance in cancer cells by regulating many apoptosis-associated proteins like XIAP and BAD.^30^ Thus, breast cancer cells resistance to taxol could be involved in activating the IGF-I/AKT or EGF/AKT pathway. Although NCOA3 did upregulate Bcl-2 expression and AKT activation, we could not exclude the possibility that it also both directly and indirectly affected other genes involved in taxol resistance. This hinted that the mechanisms by which NCOA3 enhanced taxol resistance required further investigation in the future.

Moreover, our results showed that high expression of NCOA3 identified in taxol-resistant breast cancer cells was associated with loss of miR-17 and miR-20b. MiRNAs have been identified as oncogene or tumor suppressor to regulate cancer cell functions such as cell proliferation and differentiation in breast cancer. Furthermore, growing evidence suggests that miRNAs have important roles in cancer cell resistance to chemotherapy drugs. Several studies have linked high expression of miRNAs with resistance to chemotherapeutics in breast cancers.^15^ On the other hand, loss of miRNAs was found in chemotherapy-resistant breast cancer.^17^ One recent study shows that miR-17 and miR-20b were significantly downregulated in taxol-resistant breast cancer cells by miRNA array chips.^20^ However, little is known about the roles of miR-17 and miR-20b in drugs resistance. Consistent with the previous finding, we also found both miR-17 and miR-20b were observably reduced in taxol-resistant breast cancer compared with taxol-sensitive breast cancer. Moreover, we demonstrated that miR-17 and miR-20b enhanced breast cancer cell sensitivity to taxol by directly targeting NCOA3. Our results suggested miR-17 and miR-20b could exhibit tumor-suppressor role in breast cancer.

In addition, we also showed the levels of miR-17 and miR-20b in taxol-resistant brest cancer tissues were significantly decreased relative to their adjacent normal tissues. However, no obvious differences were found between taxol-sensitive brest cancer tissues and their adjacent normal tissues. Notably, NCOA3 negatively correlated with the two miRNA was clearly upregulated in taxol-sensitive brest cancer tissues compared with normal tissues. These findings implied that downregulation of miR-17 and miR-20b could attenuate taxol resistance and failed to inhibit tumorigenesis in breast cancer. These results also suggested upregulation of NCOA3 induced mammary tumorigenesis was not involved in miR-17 and miR-20b.

MiR-17 is a member of the miR-17~92 cluster, which has crucial roles in cancer development. MiR-17 is generally upregulated in several cancer types such as breast, lung, colon and gastric cancer.^31, 32^ Moreover, previous studies indicate that miR-17 functions as a driver of cancers to promote cancer cell proliferation and migration in many types of solid tumors including melanoma and breast cancer.^32, 33^ In contrast, another study found miR-17 exhibits cancer-suppressing functions to inhibit cellular invasion and tumor metastasis in breast cancer.^33^ Moreover, overexpression of miR-17 in colorectal cancer was reported to inhibit cancer progression by targeting angiogenesis.^25^ It is previously reported that miR-20b serves as a survival and oncogenic factor in human breast cancer.^34^ However, another study has demonstrated that miR-20b exerts tumor-suppressor effects in bladder cancer and inhibits cancer cells proliferation, migration and invasion.^35^ In this study, we demonstrated the novel tumor-suppressor roles of miR-17 and miR-20b in the taxol resistance of breast cancer. All these studies suggested miR-17 and miR-20b functioned as both a driver and suppressor of cancers dependent on cellular contexts, and its function was determined by multiple factors.

In conclusion, we had identified downregulation of miR-17 and miR-20b induced taxol resistance in breast cancer by upregulating NCOA3. This study had implications for the treatment of taxol-resistant breast cancer. Our study provided useful information for the development of alternative approaches to diagnose and treat taxol-resistant breast cancer.

## Materials and Methods

### Tissues specimen information

Breast cancer samples were collected from The Cancer Center of Guangzhou Medical University (Guangzhou, China), between 2009 and 2011. A total of 55 pairs of breast cancer tissues and adjacent normal tissues were examined in the study. Patients with disease progression or recurrence 6 months or less after taxol therapy were defined as taxol resistance, whereas those without recurrence or recurrence >6 months after taxol therapy were defined as taxol sensitivity. After systemic taxol treatments, 22 taxol-sensitive patients contributed 22 of breast tumor tissues and 21 of normal tissues, and 33 taxol-resistant patients contributed 33 pairs of breast tumor and normal tissues. All of the samples were retrieved within 15 min after the surgery and immediately snap-frozen in liquid nitrogen and stored at −80 °C until used for gene expression analysis as previously described.^36^ This study was approved by the Clinical Ethics Review Board at The Cancer Center of Guangzhou Medical University and written informed consents were from all patients at their recruitment time.

### Cell culture and reagents

Human breast cancer cell lines MCF-7 and MDA-MB-231 (231) were purchased from the American Type Culture Collection (ATCC, Manassas, VA, USA). The identities of cell lines were confirmed by using DNA profiling (short tandem repeat). Taxol-resistant cell lines MCF-7/Tax1, MCF-7/Tax2, 231/Tax1 and 231/Tax2 were established from MCF-7 and 231 cells after following continuous exposure to 2 and 1 nM of Taxol (Sigma, St. Louis, MO, USA) for >12 months. All cell lines were maintained in DMEM medium supplemented with 10% fetal bovine serum (Gibco, Carlsbad, CA, USA) at 37 °C in a 5% CO_2_ humidified incubator. Cells were grown in monolayer and passaged routinely 2–3 times a week. Dimethyl sulfoxide (DMSO) was purchased from Sigma.

### Real-time PCR

Total RNA was extracted from breast cancer cell lines and patient tissues using TRIzol reagent (Invitrogen, Carlsbad, CA, USA). Following DNaseI treatment, 2 *μ*g of total RNA was reverse transcribed using cDNA synthesis kit (Bio-Rad, Richmond, CA, USA) to synthesize cDNA specimens. And then, RT-PCR analysis of gene expression was performed using 2 *μ*l of cDNA and SYBR Green Supermix (Bio-Rad, Hercules, CA, USA) as recommended by the manufacturer. RT-PCR was conducted by means of the SYBR on the CFX96 system (Bio-Rad). For miRNA, a Ploy-A tail was added to the miRNA, which was then transcribed into cDNA using a universal adaptor primer that included oligo-dT. The generated cDNA was then combined with the Uni-miR RT-PCR Primer (possess the binding site with universal adaptor primer, included in SuperScript III One-Step RT-PCR Kit with Platinum Taq (Invitrogen)) and a miRNA primer (sequence complementary to the miRNA) to complete the RT-PCR reaction. The miRNA expression was normalized using the endogenous U6 snRNA. For the primers, miR-17: 5′-GAGCCAAAGTGCTTACAGTGC-3′ (forward); miR-20b: 5′-ATGCCAAAGTGCTCATAGT-3′ (forward) and reverse-primer (Uni-miR RT-PCR primer, included in SYBR Premix Ex Taq kit (TaKaRa, Japan)). U6: 5′-CTCGCTTCGGCAGCACA-3′ (forward) and 5′-AACGCTTCACGAATTTGCGT-3′ (reverse). For *NCOA3* mRNA detection, *β*-actin was used as an internal control to normalize gene expression values. For *NCOA3* primers: 5′-CGTCCTCCATATAACCGAGC-3′ (forward) and 5′-TCATAGGTTCCATTCTGCCG-3′ (reverse). For *β-actin* primers: 5′-TTCTACAATGAGCTGCGTGTG-3′ (forward) and 5′-GGGGTGTTGAAGGTCTCAAA-3′ (reverse). The PCR was run in triplicate at 95 °C for 2 min followed by 40 cycles of 95 °C for 15 s, 56 °C for 20 s and 72 °C for 20 s. Comparative quantification was performed using the 2^-⊿⊿Ct^ method. Each sample was analyzed in triplicate.

### Plasmids and miRNAs transfection

The NCOA3 expression vector was kindly gifted from Professor Chundong Yu.^9^ MiR-17 and miR-20b mimics, inhibitors and negative control (NC) were purchased from GenePharma (Shanghai, China). Cells were trypsinised, counted and seeded into six-well plates the day before transfection to ensure 70% cell confluence on the day of transfection. Transfection of miRNA mimics/inhibitors and plasmids into cells was performed using lipofectamine 3000 (Invitrogen) in accordance with the manufacturer's advised procedure as previously described.^37^ The miRNA mimics or inhibitors and plasmids were used at a final concentration of 50 nM and 2 *μ*g, respectively. At 48 h after transfection, RT-PCR and western blotting were performed.

### Determination of cell viability and apoptosis

MTT assay was conducted to assess the cell viability as previously described.^38^ Values are expressed percent survival of the vehicle-treated control. Measurement of apoptosis was conducted by Annexin-V-FITC (fluorescein isothiocyanate)/PI (propidium iodide) analysis as described previously.^39^

### Western blot analysis

Total proteins were isolated from cells with lysis buffer (50 mM Tris, pH 7.5; 150 mM NaCl; 1% NP40; 2.5 mM sodium pyrophosphate; 0.02% sodium azide; 1 mM EGTA, 1 mM EDTA; 1 mM b-glycerophosphate; 1 mM Na_3_VO_4_; 1 mM PMSF; 1 *μ*g/ml leupeptin). The lysates were centrifuged at 12 000 r.p.m. for 30 min at 4 °C. The protein concentration was determined by Bradford dye method. Equal amounts (20–50 *μ*g) of cell extract were subjected to electrophoresis in 8–12% sodium dodecyl sulfate-polyacrylamide (SDS-PAGE) and transferred to PVDF membranes (Millipore, Darmstadt, Germany) for antibody blotting. The membranes were blocked and then incubated with NCOA3, p-AKT (ser473) antibody (Cell Signaling Technologies, Boston, MA, USA), Bcl-2 antibody (Abcam, Cambridge, UK), AKT and *β*-actin antibody (Santa Cruz Biotech, Santa Cruz, CA, USA) overnight at 4 °C. Subsequently, the membranes were incubated with a HRP-conjugated anti-mouse or anti-rabbit secondary antibody (Protein Tech Group, Chicago, IL, USA) at room temperature for 1 h. The protein bands were visualized using an enhanced chemiluminescence reagent kit (GE Healthcare, Munich, Germany), according to the manufacturer's instructions.^40^

### miRNA target prediction

The analysis of NCOA3 predicted targets was performed using the algorithms TargetScan (http://targetscan.org/), PicTar4 (http://pictar.mdc-berlin.de/) and miRDB (http://www.mirdb.org/miRDB/), miRWalk (http://zmf.umm.uni-heidelberg.de/apps/zmf/mirwalk/), miRanda (http://www.microrna.org/microrna).^41^

### Dual-luciferase activity assay

The human 3′-UTR of *NCOA3* genes were amplified by PCR and cloned into the *Xba*I site of the pGL3-Control vector (Promega, Madison, WI, USA), downstream of the luciferase gene, to generate the vector pGL3-NCOA3. For luciferase assay, the 293 T and 231/Tax1 cells were cultured in 24-well plates and transfected with 500 ng of pGL3-NR5A2, pGL3-CREB1 or pGL3-control vector along with 50 pmol of miR-17 mimics and miR-20b mimics or NCs, respectively. Transfection of miRNAs was carried out using Lipofectamine 3000 in accordance with the procedure of the manufacturer (Life Technologies, Carlsbad, CA, USA). At 24 h after transfection, firefly luciferase activity was measured using the Dual-Luciferase Reporter Assay (Promega) as previously described.^36^ The above experiment was repeated at least three times.

### Animal experiment

Athymic BALB/c nude mice (4–6 weeks old) were obtained from Si-Lai-Ke-Jing-Da Experimental Animal Co. Ltd (Changsha, China). All of the procedures of animal experiments were performed according to approved protocols and in accordance with the guidelines of the Guide for the Care and Use of Laboratory Animals (Institute of Laboratory Animal Resources, Commission on Life Sciences, National Research Council). It was approved by the Institutional Animal Care and Use Committee of The Cancer Center of Guangzhou Medical University. MCF-7/Tax1/NC, 231/Tax1/NC, MCF-7/Tax1/miR-17, 231/Tax1/miR-17, MCF-7/Tax1/miR-20b and 231/Tax1/miR-20b cells with vector and stably overexpressed miR-17 and miR-20b were constructed by lentivirus (Genepharma). For MCF-7 xenograft, mice were anesthetized and implanted subcutaneously 17*β*-estrogen pellets (0.18 mg/60-day release) (Innovative Research of America, Sarasota, FL, USA) above the shoulders using a 10-gauge trochar.^42^ After 7 days, MCF-7 cells were subcutaneously injected into the mice with 17*β*-estrogen pellets. In brief, cells (for MCF-7: 8 × 10^6^ cells; for 231: 4 × 10^6^ cells) were suspended in 0.2 ml of 50% phenol red-free Matrigel (Becton Dickinson Bioscience, San Jose, CA, USA) mixed with serum-free RPMI/1640 media and were injected into the flanks of female BALB/c nude mice without anesthesia (4–6 weeks old, *n*=6 per group), which were maintained under pathogen-free conditions. Fifteen days (MCF-7 cells) or 25 days (231 cells) after tumor cells implantation, mice were treated with taxol and health of mice was checked every day. In xenograft mice, individual treatments of taxol at 20 mg/kg were administered once every 5 days for a total of five courses. Tumor volume was measured once every 3 days by using calipers as indicated at each time point. The tumor volume was estimated by the following formula: length × width × width × 3.14/6. The mice whole body weight was measured once every 3 days as indicated time point. All mice were killed by intraperitoneal injection of 200 mg/kg pentobarbital at the end of the experiment.

### Statistical analysis

Results were expressed as mean±S.D. Differences between groups were estimated using the Student's *t*-test. A level of *P*<0.05 was considered to be significant. The relationships between miR-17 or miR-20b and NCOA3 expression level were analyzed by correlation coefficients and linear regression analysis.^43^ All analyses were performed using SPSS16.0 software (IBM, Armonk, NY, USA) and a two-tailed value of *P*<0.05 was considered statistically significant.

## Abbreviations

miRNAs: microRNAs
NCOA3: nuclear receptor coactivator 3
microRNA-17: miRNA-17
microRNA-20b: miRNA-20b
siRNA: small interfering RNA
3′-UTR: 3′-untranslated region
