# Supplementary material for: Decreased expression of microRNA-17 and microRNA-20b promotes breast cancer resistance to taxol therapy by upregulation of NCOA3
Source: Cell Death Dis. 2016 Nov 10;7(11):e2463–. doi: 10.1038/cddis.2016.367 (PMC5260895; doi:10.1038/cddis.2016.367)
Supplement: Supplementary Table S1 [file cddis2016367x1.doc]

**Table S1** Clinical information of the 55 patients included in this study

| **Characteristics** | **Patients with taxol sensitivity** | | | **Patients with taxol resistance** | |
| --- | --- | --- | --- | --- | --- |
|  | **Number (%)** | | **Number (%)** | | |
| **Age 55 (25-72) years**  <35  35-55  >55  **Histology**  Infiltrating ductal carcinoma  Infiltrating (mixed) carcinoma  Others  **TNM stage**  II  IIIA  IIIB-IV  **Estrogen receptor status**  Negative  Positive  **Progesterone receptor status**  Negative  Positive  **Her2 status**  Negative  Positive | **22**  **7**  **10**  **5**  **10**  **8**  **4**  **6**  **10**  **6**  **13**  **9**  **16**  **6**  **9**  **13** | **31.8**  **45.4**  **22.7**  **45.4**  **36.4**  **18.2**  **27.3**  **45.5**  **27.3**  **59.1**  **40.9**  **72.7**  **27.3**  **40.9**  **50.1** | | **33**  **6**  **9**  **18**  **9**  **16**  **8**  **6**  **8**  **19**  **18**  **15**  **20**  **13**  **19**  **14** | **18.2**  **27.3**  **54.5**  **27.3**  **48.5**  **24.2**  **18.2**  **24.2**  **57.6**  **54.5**  **45.5**  **60.6**  **39.4**  **57.6**  **42.4** |

**Supplementary materials and methods**

**Caspase-3 activity assays**

Assays of caspase-3 activity were carried out by using caspase-3 assay kit (abcam, ab39383) according to the manufacturer’s protocol as described previously [1].

**Sub-G1 assays**

Measurement of sub-G1 was conducted by propidium iodide (PI) analysis. Briefly, single-cell suspensions were fixed in ice-cold 70% ethanol overnight, washed twice in PBS. And then cells were incubated with PI staining solution [0.1% Triton X-100 (Sigma-Aldrich), 50 μg/mL PI (Sigma-Aldrich), and 100 μg/ml DNase-free RNase A (Sigma-Aldrich) in PBS] for at least 30 min in dark at RT, and monitored with the FL3 channel in a FACS CaliburTM flow cytometer. The Sub G1 peak was utilized as a measure of apoptosis. The cell cycle data was analyzed with FlowJo V7 software (Tree Star, Oregon, US)

**Supplementary reference**

[1] Lou YF, Zou ZZ, Chen PJ, Huang GB, Li B, Zheng DQ *et al.*. Combination of gefitinib and DNA methylation inhibitor decitabine exerts synergistic anti-cancer activity in colon cancer cells. *PloS One* 2014; **9:** e97719.

**Supplementary** **figure legend**

**Figure S1** NCOA3 mRNA levels were significantly up-regulated in breast cancer tissues. (**A** and **B**) RT-PCR was performed to detect the expression of NCOA3 in 21 pairs of tumor and adjacent normal tissues from taxol-sensitive ptients (**A**), 33 pairs of tumor and adjacent normal tissues from taxol-resistant patients (**B**). β-actin was used as an internal control. Data represent means of three determinations.

**Figure S2** NCOA3 expression in different breast cell lines. (A) NCOA3 expression in different triple-negative breast cancer (TNBC) and Non-TNBC breast cell lines was measured by Western blot analysis. Actin was used as a loading control. (B) Relative NCOA3 protein levels compared to SK-BR-3 cells were quantified using Image J software. The experiments were repeated three times. Data represent mean ± SD.

**Figure S3** Depletion of NCOA3 increased BAX and decreased MCL-1 levels in MCF-7/Tax1 cells. MCF-7/Tax1were transfected with NC and NCOA3 siRNA for 48 h. Western blot was performed to detect the indicated protein expression. Data were from three independent experiments.

**Figure S4** Relative expression of miR-20b and miR-17 in breast tumor tissues. (**A** and **B**) RT-PCR was performed to detect the expression of miR-20b (**A**) and miR-17 (**B**) in 21 pairs of tumor and adjacent normal tissues from taxol-sensitive ptients. (**C** and **D**) RT-PCR was performed to detect the expression of miR-20b (**C**) and miR-17 (**D**) in 33 pairs of tumor and adjacent normal tissues from taxol-resistant patients. U6 was used as an internal control. Data represent means of three determinations.

**Figure S5** Both miR-17 and miR-20b enhances taxol-induced apoptosis in breast cancer cells. (**A-C**) MCF-7/Tax1 and 231/Tax1 were transfected with miR-17 or miR-20b mimics or negative control (NC) for 48 h, respectively. (**D** and **E**) MCF-7 and 231 cells were transfected with miR-17 or miR-20b inhibitors or NC for 48 h, respectively. After 8 h, cells were treated with indicated dose of taxol (Tax) for additional 48 h. Cell apoptosis was assessed by sub-G1 assay by flow cytometry. Columns, means of three determinations; bars, SD. *, *P* < 0.05; **, *P* < 0.01; ***, *P* < 0.001, compared with NC-treated cells.

**Figure S6** Both miR-17 and miR-20b enhances taxol-induced caspase 3 activities in breast cancer cells. (**A** and **B**) MCF-7/Tax1 and 231/Tax1 were transfected with miR-17 or miR-20b mimics or negative control (NC) for 48 h, respectively. (**C** and **D**) MCF-7 and 231 cells were transfected with miR-17 or miR-20b inhibitors or NC for 48 h, respectively. After 8 h, cells were treated with indicated dose of taxol (Tax) for additional 48 h. The caspase 3 activities were quantified as described under Methods. Columns, means of three determinations; bars, SD. *, *P* < 0.05; **, *P* < 0.01; ***, *P* < 0.001, compared with NC-treated cells.

**Figure S7** Overexpression of NCOA3 reverses reduction of NCOA3 mRNA levels by miR-17 and miR-20b in taxol-treated breast cancer cells. (**A** and **B**) MCF-7/Tax1 (**A**) and 231/Tax1 (**B**) cells were cotransfected with nagative control (NC) and miR-17 or miR-20b mimics along with control (Ctr) or NCOA3 vectors. After 48 h, relative expression of NCOA3 were determined by RT-PCR. β-Actin was used as an internal control. Columns, means of three determinations; Bars, SD; **, *P* < 0.01; ***, *P* < 0.001, compared with the cells treated with NC mimics plus CTR vector.
