# Supplementary figures and images for: Decreased expression of microRNA-17 and microRNA-20b promotes breast cancer resistance to taxol therapy by upregulation of NCOA3
Source: Cell Death Dis. 2016 Nov 10;7(11):e2463–. doi: 10.1038/cddis.2016.367 (PMC5260895; doi:10.1038/cddis.2016.367)

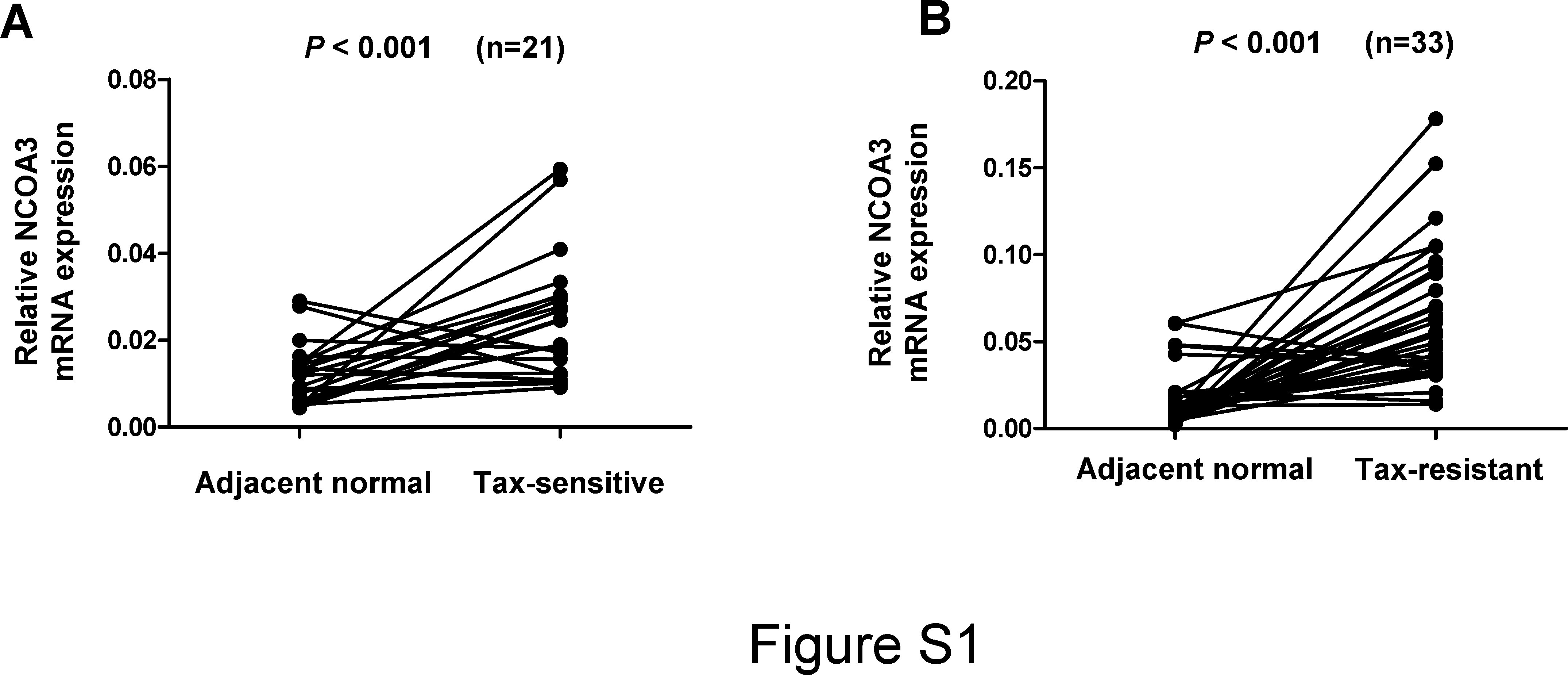

Supplement: Supplementary Figure S1 [file cddis2016367x2.tif]

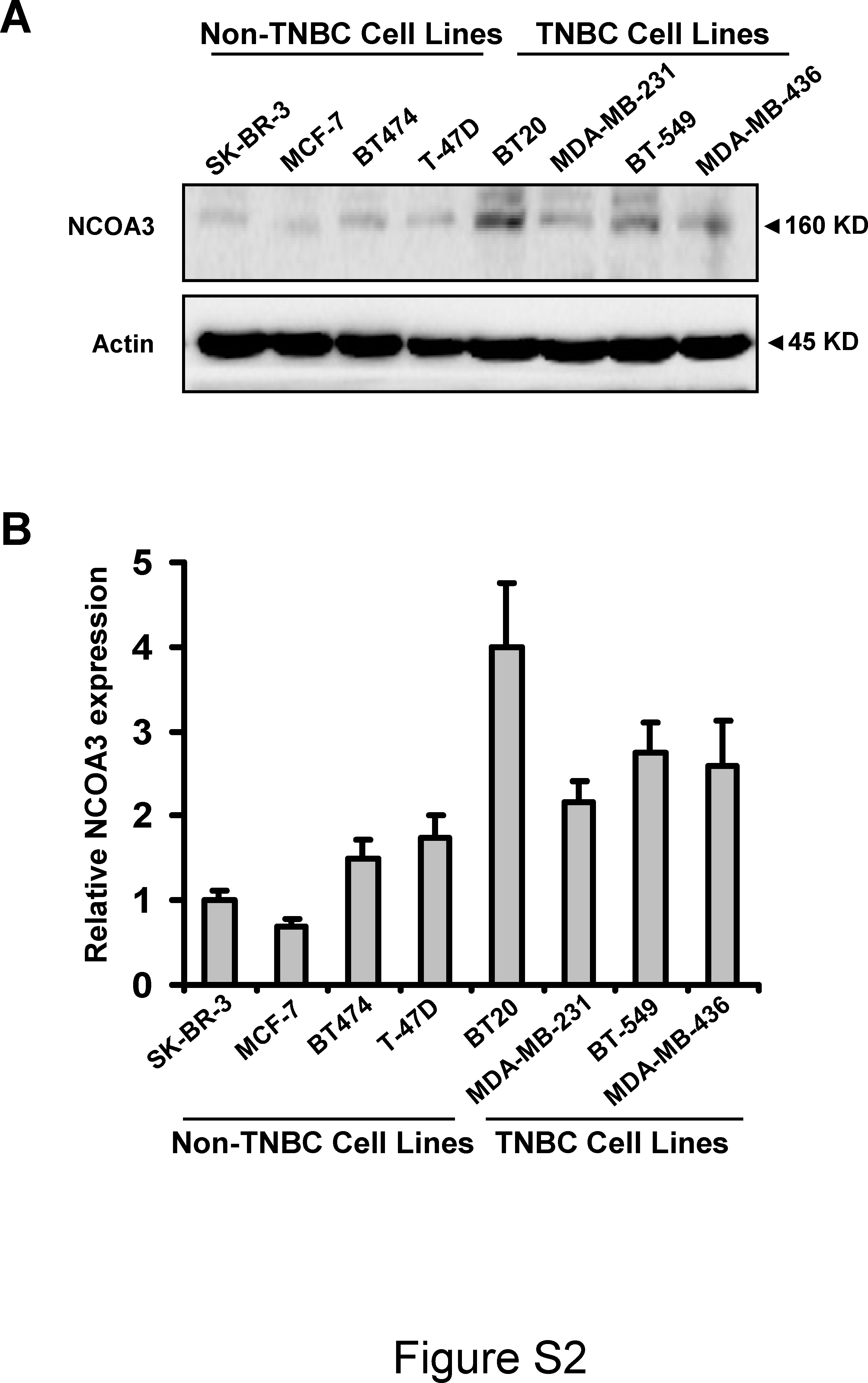

Supplement: Supplementary Figure S2 [file cddis2016367x3.tif]

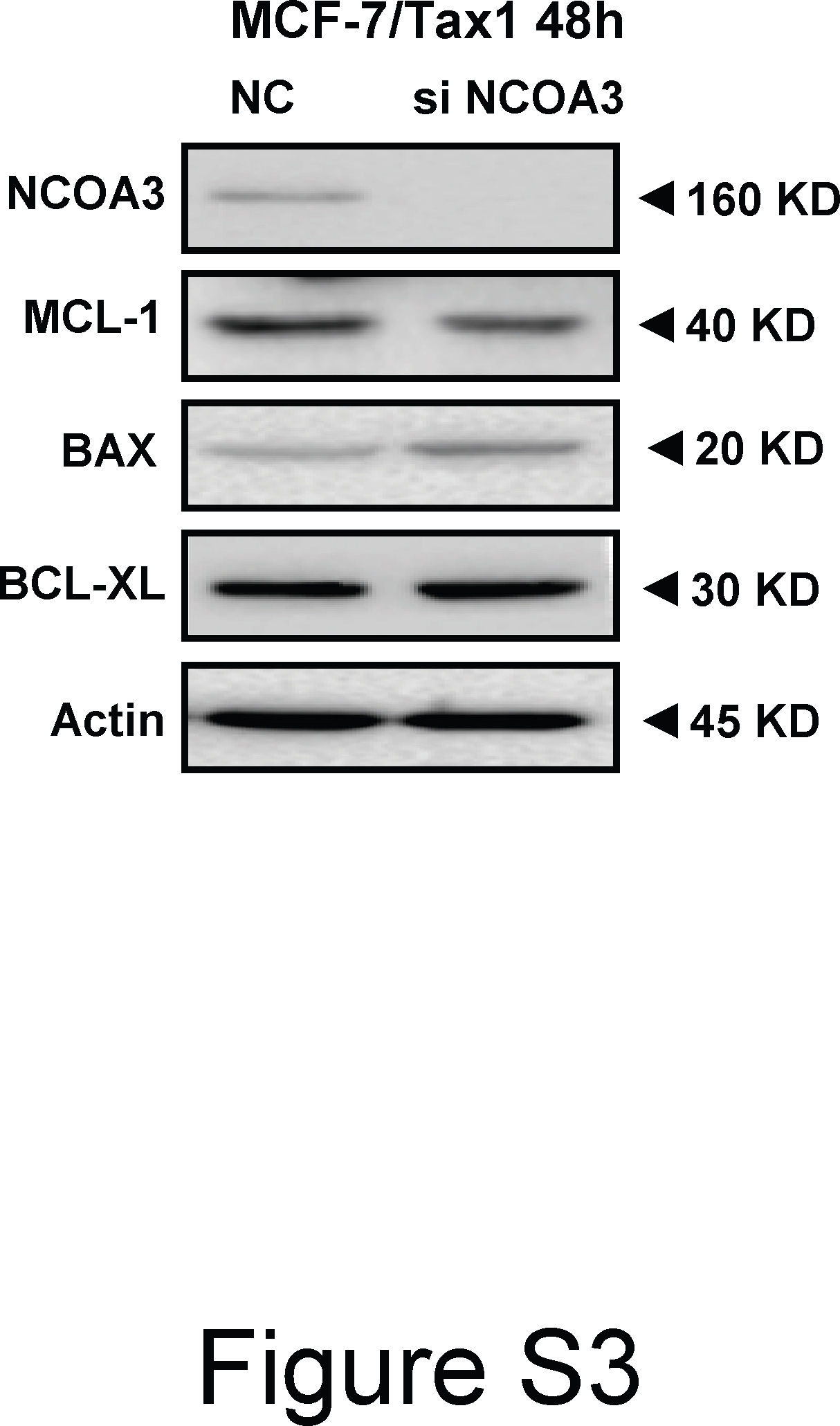

Supplement: Supplementary Figure S3 [file cddis2016367x4.tif]

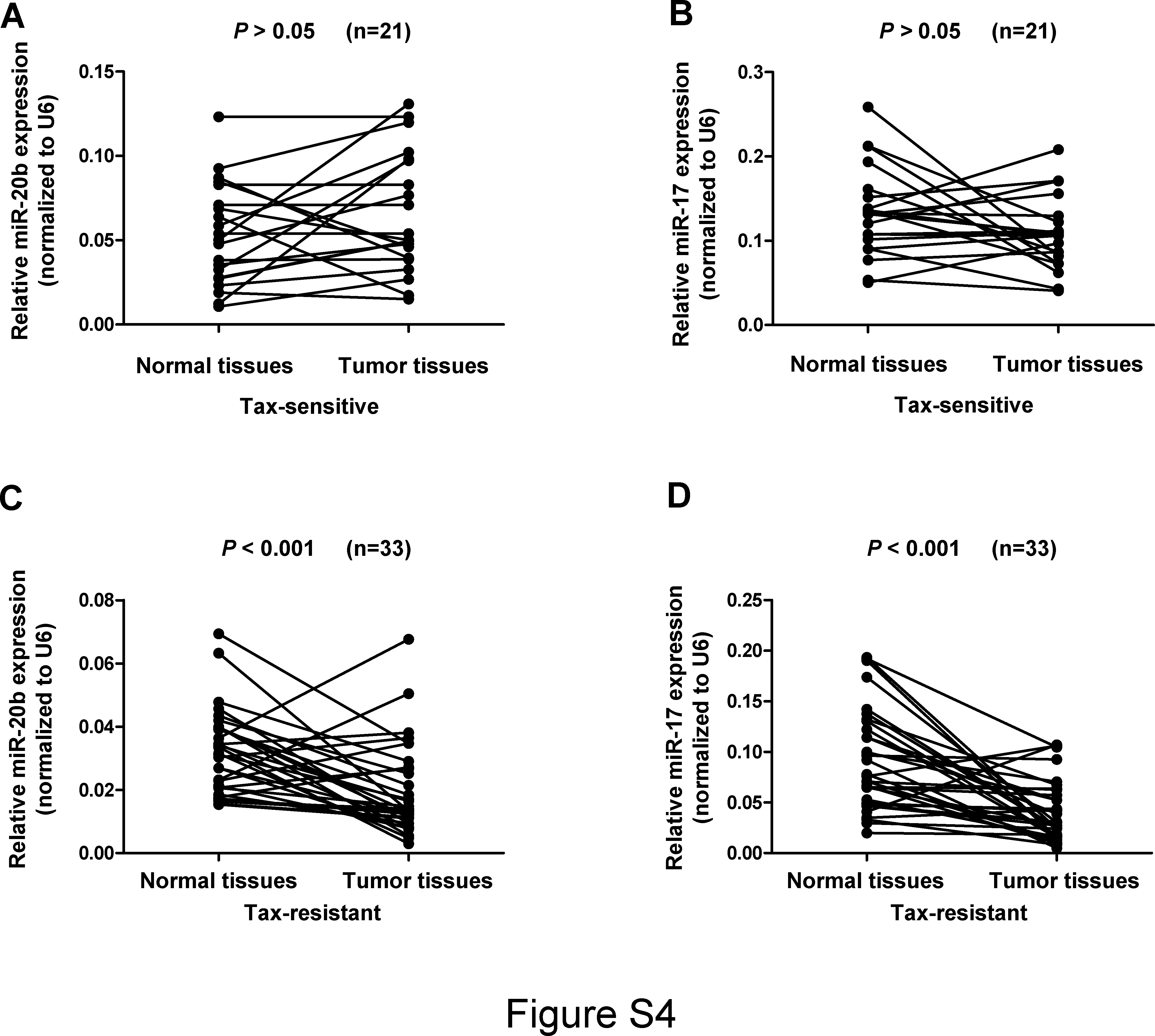

Supplement: Supplementary Figure S4 [file cddis2016367x5.tif]

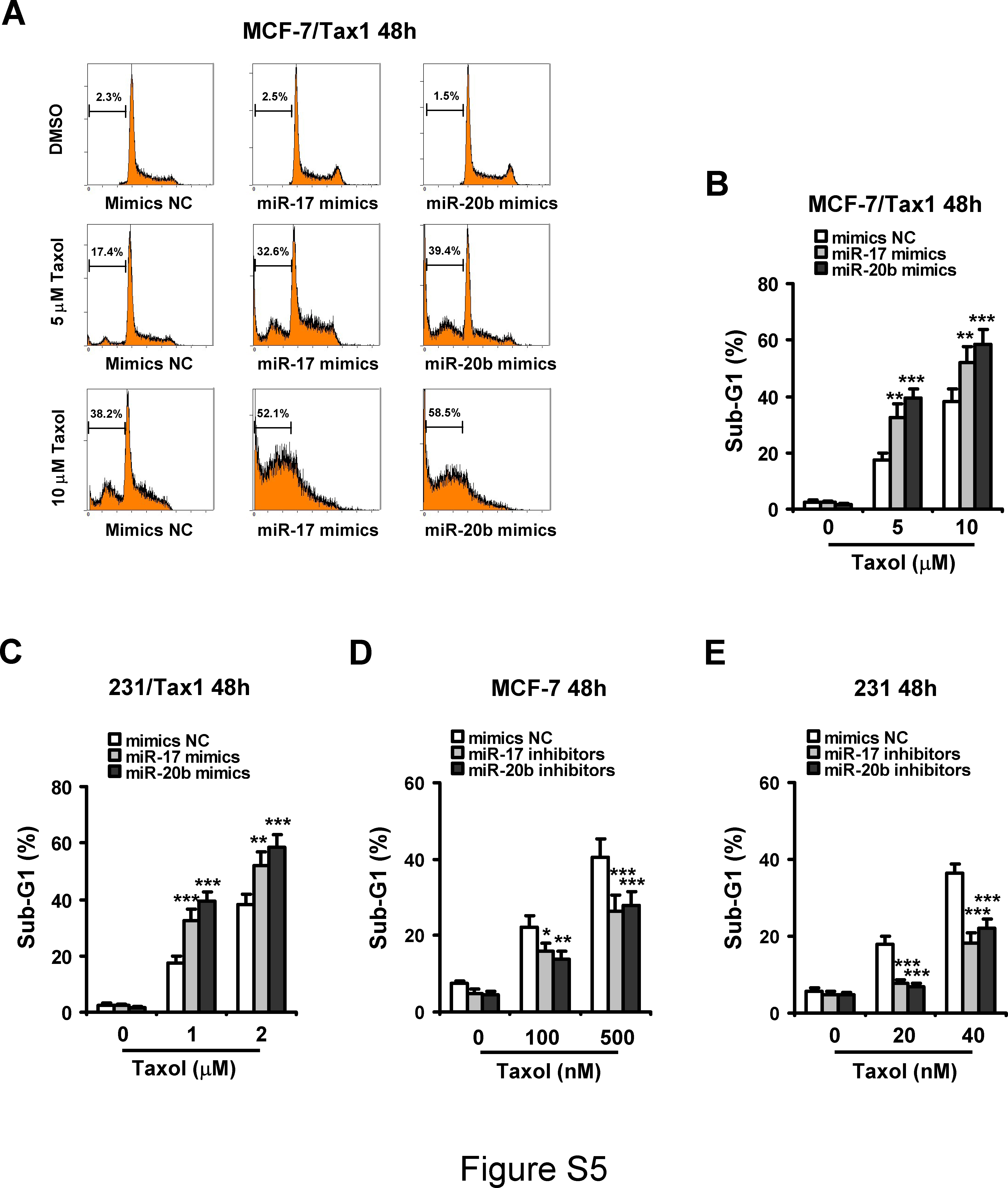

Supplement: Supplementary Figure S5 [file cddis2016367x6.tif]

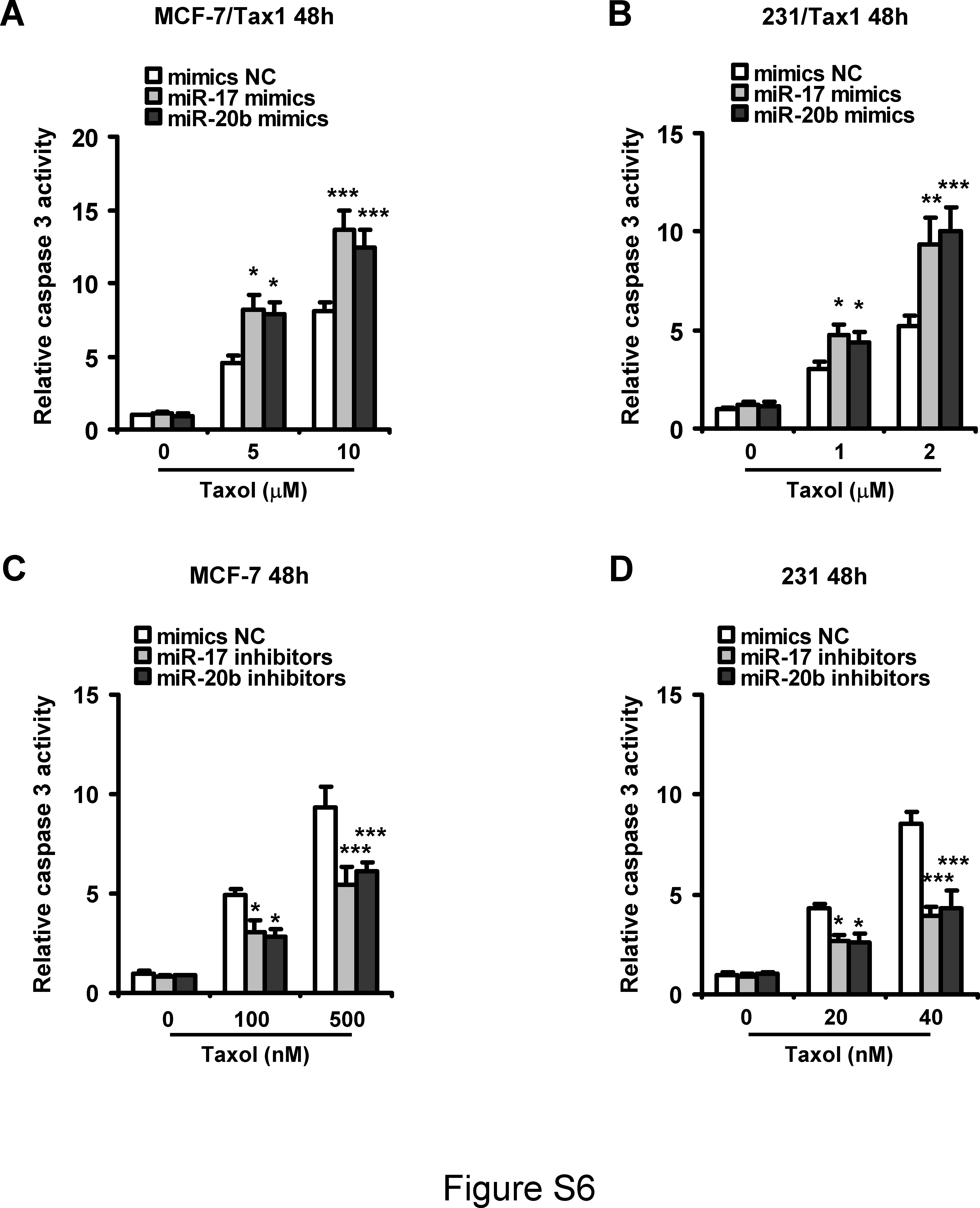

Supplement: Supplementary Figure S6 [file cddis2016367x7.tif]

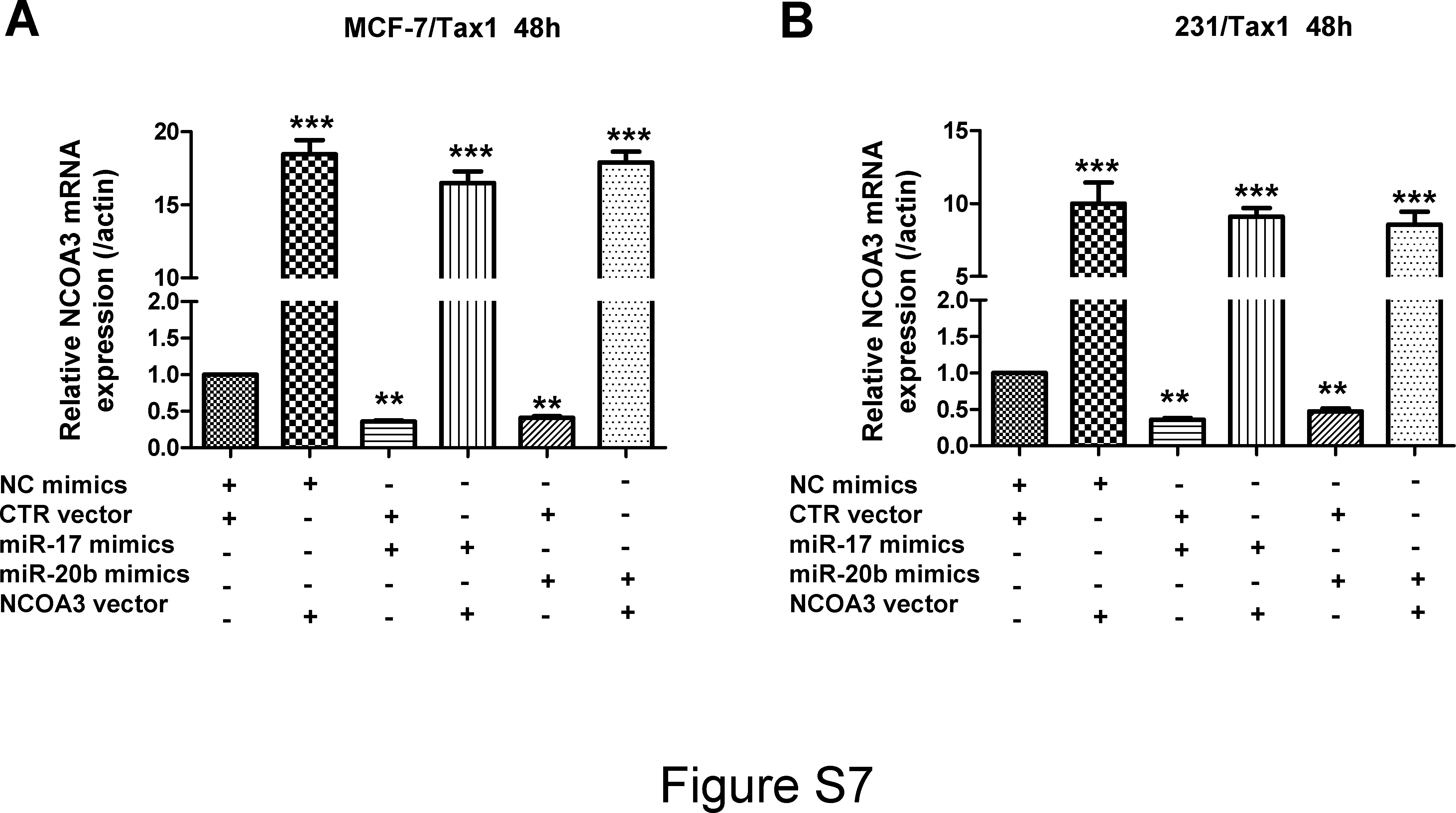

Supplement: Supplementary Figure S7 [file cddis2016367x8.tif]
